# Skin Calorimetry of the Vastus Lateralis During Incremental Exercise: Thermal Analysis and Modeling

**DOI:** 10.3390/bios16090471

**Published:** 2026-08-27

**Authors:** Pedro Jesús Rodríguez de Rivera, Miriam Rodríguez de Rivera, Fabiola Socorro, Eduardo Garcia-Gonzalez, Elisabetta De Nigris, Jose A. L. Calbet, Manuel Rodríguez de Rivera

**Affiliations:** 1 Department of Physics, Universidad de Las Palmas de Gran Canaria (ULPGC), 35017 Las Palmas de Gran Canaria, Spain; fabiola.socorro@ulpgc.es (F.S.); manuel.rguezderivera@ulpgc.es (M.R.d.R.); 2 Cardiology Service, Hospital Universitario Marqués de Valdecilla, 39008 Santander, Spain; miriam.rodriguezderivera@scsalud.es; 3 Department of Physical Education and Research Institute of Biomedical and Health Sciences (IUIBS), University of Las Palmas de Gran Canaria, 35016 Las Palmas de Gran Canaria, Spain; eduardo.garcia@ulpgc.es (E.G.-G.); elisabetta.de101@alu.ulpgc.es (E.D.N.); lopezcalbet@gmail.com (J.A.L.C.); 4 School of Kinesiology, Faculty of Education, The University of British Columbia, Vancouver, BC V6T 1Z1, Canada

**Keywords:** sports medicine sensors, skin heat flow, skin calorimeter, skin calorimetry, thermal simulations

## Abstract

In this study, we examined the thermal response of the vastus lateralis muscle using a skin calorimeter designed for localized measurements. Five healthy young male participants (20–23 years old) performed an incremental test on a cycle ergometer at 20 W·min^−1^ until exhaustion. The calorimeter’s thermostat was maintained at 30 °C. Ambient temperature was 23 ± 1 °C, and relative humidity ranged from 55% to 60%. The measured heat flow was modeled using functions relating mechanical power output to the thermal response. The model included the exercise phase, recovery, and sweat evaporation. The proposed model accurately reproduced the experimental data and supported a physiological interpretation of the main thermal effects. Two major contributions were identified and physiologically interpreted as muscle warming due to increased metabolic activity and a cooling effect likely linked to changes in blood perfusion. For an area of 2 × 2 cm^2^ and an incremental exercise of 20 W·min^−1^, the following values were obtained: (1) a resting heat loss of 150 ± 20 mW; (2) an exponential increase due to exercise of 0.4 ± 0.1 mW·W^−1^, with a time constant of 1.0 ± 0.3 min; and (3) a negative blood-flow contribution described by a function that, in the steady state, has an amplitude of −20 ± 9 mW. Since only five participants were included, correlations with anthropometric variables were treated as exploratory consistency checks. These results demonstrate the usefulness of the calorimeter for decomposing and quantifying muscle thermal dynamics during exercise.

## 1. Introduction

The measurement of heat flux and its spatial distribution in active tissues provides valuable insights into muscle metabolism, mechanical efficiency, and heat transfer mechanisms during exercise. Heat generated during muscle contraction is an inevitable by-product of metabolism. How this heat is conducted to the body surface depends on blood perfusion, tissue thermal properties, and the subject-specific tissue distribution [1]. Indirect calorimetry remains the gold standard for assessing whole-body metabolic energy expenditure under controlled conditions [2,3], while local sensors are becoming increasingly popular for characterizing regional thermal responses in the skin.

The most commonly used sensors for studying local thermal dynamics include contact and remote temperature sensors [4,5]. In this context, body heat storage and mean body temperature during thermal stress can be assessed using temperature probes and whole-body direct calorimetry [6], while infrared thermography enables non-invasive detection of local thermal anomalies [7]. Invasive approaches, including catheter-based intravascular temperature measurement, thermodilution flow assessment, and intramuscular multisensor temperature probes, have also been used to evaluate limb hemodynamics and local responses during exercise [8,9]. Heat flux sensors have also been used in this field because they provide a direct estimate of local heat exchange, although their measurements can be difficult to interpret [10]. In the field of local contact measurements, our group has developed a compact contact calorimeter to measure temperature, heat flow, thermal resistance and heat capacity of localized 2 × 2 cm^2^ skin areas [11].

Among non-invasive thermal monitoring techniques, the dual heat flux (DHF) method estimates core body temperature using two heat conduction paths with different thermal resistances [12,13]. Although this method has proved useful for estimating deep tissue temperature, it is not designed to directly measure local heat flow or characterize the dynamic thermal response of superficial tissues. In contrast, the skin calorimeter used in this study directly measures local heat flow with a thermopile under controlled thermal conditions. This allows muscle heat production and its evolution during exercise to be analyzed. Other techniques, such as thin film thermal sensors [14,15], have been developed to measure the thermal conductivity of the skin, with promising results. These studies have shown that skin thermal conductivity depends on water content and blood perfusion, both of which are influenced by changes in skin temperature.

Despite advances in cutaneous heat measurements, the integration of dynamic thermal analysis and mathematical modeling for human muscles during exercise remains underdeveloped. In this context, the Pennes bioheat equation [16,17] provides a useful framework for describing local heat transfer in the human body and has been widely applied to problems involving altered heat flow in pathological tissues and to thermal treatments such as therapeutic hyperthermia [18,19,20].

In a previous study [21], we used our skin calorimeter [11] to measure and analyze the thermal response of the rectus femoris muscle during a constant moderate-intensity exercise protocol at 80 W on a stepper, performed by a single young participant. Multiple measurements were conducted at different thermostat temperatures. Based on these measurements, a mathematical model was proposed to relate the mechanical work performed by the participant to the locally measured heat flow. In all cases, the model parameters could be identified and physically interpreted, and the model was consistent with the Pennes bioheat equation (two exponential terms) [22], thereby fulfilling the main objective of that study. However, because the measurements were performed during exercise, an additional linear–exponential term was introduced to account for the thermal response associated with sustained mechanical work. This formulation enabled identification of the process, although its scope was limited by its reliance on a single participant and a single exercise protocol.

Building on the previous proof-of-concept study [21], which was limited to one participant and a constant-load exercise protocol, the present work extends the approach to five young male participants aged 20–23 years performing an incremental cycling test to exhaustion, with a workload increase of 20 W·min^−1^, a commonly used protocol in exercise physiology and cardiopulmonary exercise testing [23,24]. The continuously increasing workload produces a more complex thermal response and allows the model to be evaluated under more demanding physiological conditions. The measurement site was changed from the rectus femoris to the vastus lateralis because this muscle is strongly involved in cycling and provides a suitable superficial location for measuring local muscle heat flow. All measurements were performed at a constant skin calorimeter thermostat temperature of 30 °C. Heat flow responses were analyzed during exercise and recovery, including the local cooling effect associated with sweat evaporation. Based on these data, a mathematical model was developed to describe the heat flow response from the initial resting state through exercise and recovery. The main objective of this work was to evaluate the model’s ability to reproduce the measured heat flow and to provide proposed physiological interpretations of the estimated parameters.

## 2. Materials and Methods

### 2.1. Skin Calorimeter Experimental Setup

**Figure 1:**
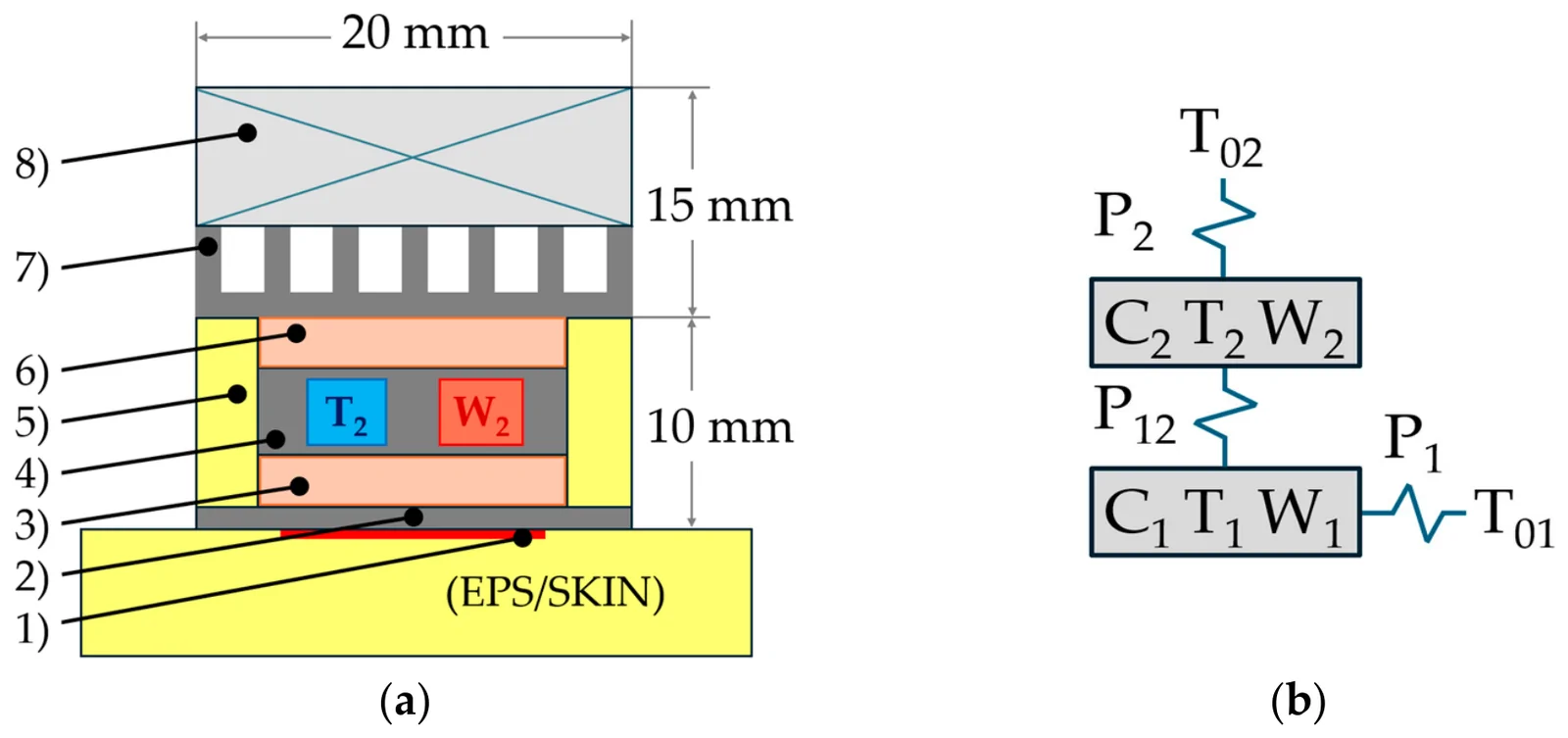
(**a**) Simplified cross–section of the skin calorimeter: 1) calibration resistor, 2) measuring plate, 3) measuring thermopile, 4) thermostat, 5) lateral EPS insulation, 6) Peltier module, 7) heat sink, 8) fan. (**b**) Calorimetric model. *C*_1_: measuring plate + involved skin region, *C*_2_: thermostat.

Calorimetric measurements were obtained using two skin calorimeters developed in our laboratory. These devices were designed to quantify heat flow dissipated from localized skin regions and have been described in detail in previous studies [11,21]. A simplified cross–section of the device is shown in Figure 1a. The device essentially consists of a measuring thermopile (module ET2065F2A131211W2.25 by Laird, Wirral, UK, part 3 in Figure 1a) placed between an aluminum measuring plate (part 2 in Figure 1a, with a sensing area of 4 cm^2^) and a thermostat (part 4 in Figure 1a). The thermostat dissipates power *W*_2_, calculated by a PID controller, to maintain its temperature at the programmed value, *T*_2_. The thermostat requires a cold source, consisting of a Peltier module (of the same type as the measurement one), a heat sink, and a fan (parts 6, 7, and 8 in Figure 1a, respectively). The thermostat temperature can be programmed over a wide range; however, in human heat flow measurements, it is usually maintained at a constant value between 25 and 35 °C. For calibration, an electrical resistor is attached to the measuring plate (part 1 in Figure 1a) while the calorimeter is placed on an insulating EPS surface. Under these conditions, the heat generated by the resistor is assumed to be transferred to the calorimeter with negligible losses. Lateral thermal insulation minimizes convective and radiative losses (part 5 in Figure 1a).

To relate the heat flow passing through the calorimeter to the recorded signals, a simple two-element calorimetric model was used (Figure 1b) [25,26]. The first element represents the measurement region, whose heat loss, *W*_1_, is to be determined. This region comprises the calorimeter measuring plate (part 2 in Figure 1a) and the skin region involved in the measurement. The second element represents the thermostat, which dissipates the power *W*_2_ required to maintain its temperature *T*_2_, at the programmed value. Each element is characterized by its heat capacity, *C*_1_ and *C*_2_, and its temperature, *T*_1_, and *T*_2_, respectively. Both elements are coupled through the measuring thermopile, which has a thermal conductance *P*_12_. Consequently, the measuring thermopile generates a calorimetric signal *y*, proportional to the temperature difference between the two elements through the Seebeck coefficient, *k:*

(1) $$y(t)=k(T_{1}-T_{2}),$$

where *k* is the Seebeck coefficient of the measuring thermopile, *y*(*t*) is the calorimetric signal and *T*_1_, *T*_2_ are the domain temperatures.

The first element is connected to its immediate surroundings, at temperature *T*_01_, through the lateral insulation (part 5 in Figure 1a), which has a thermal conductance *P*_1_. The second element is connected to the cold focus, at temperature *T*_02_, through a thermal conductance *P*_2_. Thus, the conductive heat transfer equations between these domains can be written as:

(2) $$W_{1}=\frac{C_{1}}{k}\frac{dy}{dt}+\frac{P_{1}+P_{12}}{k}y+C_{1}\frac{dT_{2}}{dt}+P_{1}(T_{2}-T_{01})$$

(3) $$W_{2}=-\frac{P_{12}}{k}y+C_{2}\frac{dT_{2}}{dt}+P_{2}(T_{2}-T_{02})$$

where each equation corresponds to the power balance of each domain of the model.

The external temperatures of both elements (*T*_01_ and *T*_02_) are affected by the ambient temperature *T*_0_, and, indirectly, by the supply current of the cooling Peltier element, *I_pel_*:

(4) $$T_{01}=T_{0}+f(I_{Pel});T_{02}=T_{0}+g(I_{Pel})$$

(5) $$\Delta T_{012}=T_{01}-T_{02}=\sum_{i=0}^{2}\alpha_{i}I_{pel}^{i}$$

It should be noted that the external temperatures *T*_01_ and *T*_02_ differ from the ambient temperature, *T*_0_. This is common in locally applied thermal instruments and results from heating or cooling of the surroundings close to the sensor [27].

#### 2.1.1. Calibration

**Figure 2:**
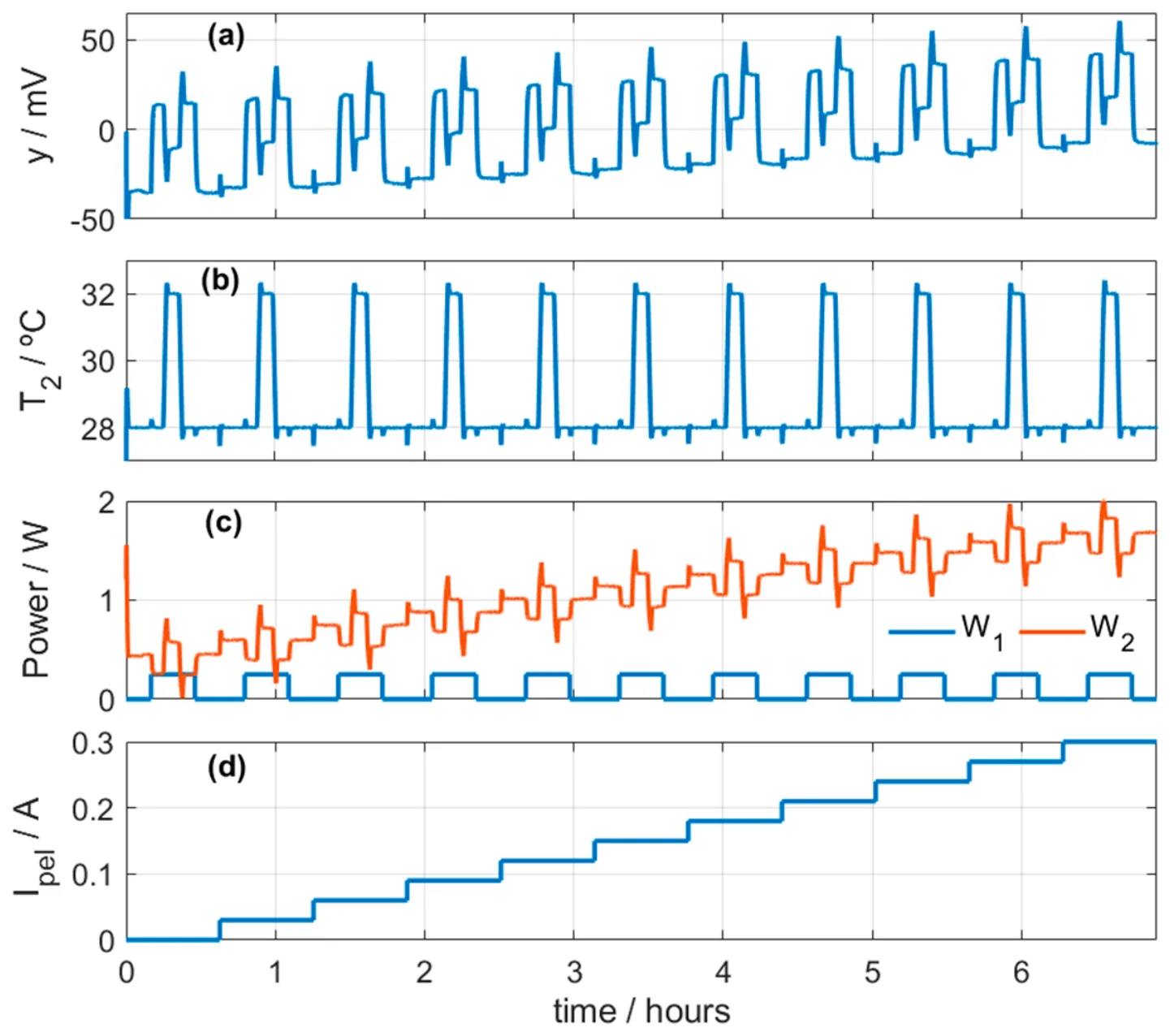
Joule calibration test. (**a**) Calorimetric signal *y*, (**b**) thermostat temperature *T*_2_, (**c**) Powers dissipated in the measurement plate *W*_1_, and in the thermostat *W*_2_ and (**d**) Peltier current *I_pel_*.

For calibration of the calorimeters, a set of tests was designed to cover a broad operating domain of the instrument by varying the power *W*_1_ dissipated in the calibration resistor (part 1 in Figure 1a), the thermostat temperature *T*_2_, and the Peltier current *I_pel_*. Two calibration ranges were explored for *W*_1_: 0–150 mW and 0–250 mW. Each calibration test consisted of a sequence of 11 consecutive measurements performed at different values of *I_pel_* (from 0 to 0.3 A) and *T*_2_ (from 28 to 32 °C). This test was repeated 16 times on different days. Figure 2 shows a representative test, displaying the calorimetric signal *y*, the thermostat temperature *T*_2_, the dissipated powers *W*_1_ and *W*_2_, and the Peltier current *I_pel_*.

The parameters of the model equations (Equations (2)–(5)) were determined from these experimental measurements. The identification procedure consisted of minimizing an error criterion defined as a linear combination of the root-mean-square errors (RMSE) between the experimental and calculated curves for both the calorimetric signal and the thermostat temperature. The calculated curves were obtained from the model using the experimental powers (*W*_1_) and (*W*_2_) as input variables. The minimization algorithm was based on the Nelder–Mead simplex method, in the version adapted by Lagarias et al. [28,29], using the MATLAB (version R2024a) function fminsearch [30].

The estimated parameters of the calorimetric model (Equations (2) and (3)), obtained from the calibration, are shown in Table 1. The parameters of the thermal model describing the external temperature (Equations (4) and (5)) are presented in Table 2. The mean RMSE was approximately 0.6 mV for the calorimetric signal and 0.025 °C for the thermostat temperature, representing about 1% and 0.6% of their respective maximum variations.

#### 2.1.2. Heat Flow Determination

For thermopile-based heat flux sensors, a direct relationship between the electrical signal and the measured heat flux is often assumed.

However, this assumption is not always valid: the measured heat flux differs from the undisturbed skin heat flux, depending on the ambient conditions and the heat exchanges with the sensor itself [31,32]. This makes comparisons among local heat loss measurements particularly difficult. For this reason, our calorimeter incorporates a thermostat, which allows the thermal perturbation produced by the sensor itself to be controlled and the measurements to be standardized [33].

The calorimetric model used in this work includes the main heat transfer paths, allowing the heat flow measured *W*_1_ to be determined in three steps:

1. *T*_02_ is calculated from the Equation (3);
2. *T*_01_ is calculated from Equations (4) and (5);
3. *W*_1_ is calculated from the Equation (2).

**Figure 3:**
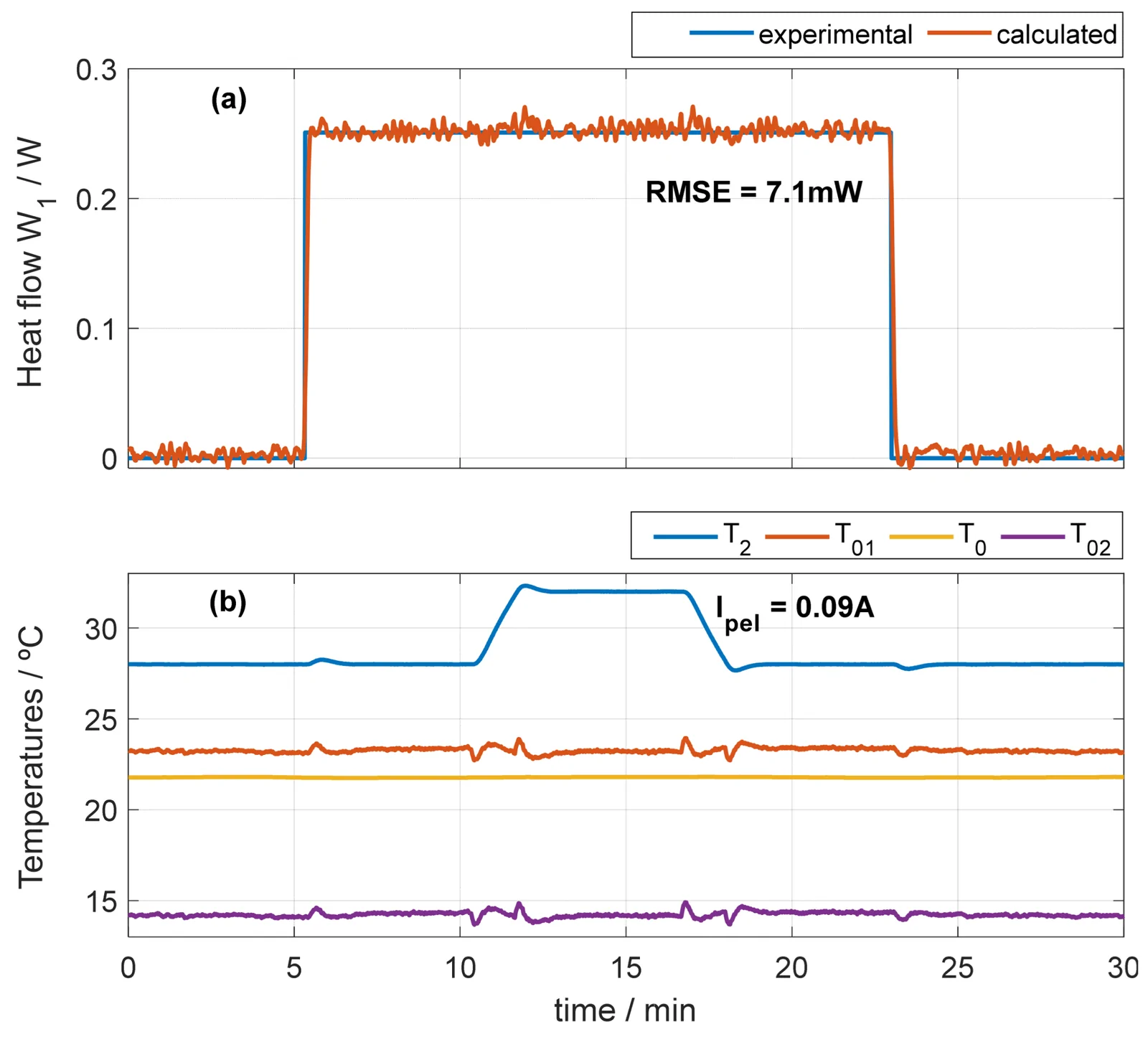
(**a**) Heat flow *W*_1_ for a Joule dissipation measurement for a Peltier current of *I_pel_* = 0.09 A. Experimental and calculated curves are shown. (**b**) Thermostat temperature *T*_2_, external temperatures, *T*_01_ and *T*_02_ and ambient temperature *T*_0_.

Figure 3 illustrates an example of heat flow reconstruction for a Joule dissipation measurement, with an RMSE of 7.1 mW, equivalent to 2.8% of the maximum value. In calorimetry, errors below 5% in determining thermal power are considered acceptable [34], especially for non-differential instruments. All heat-flow measurements obtained with the skin calorimeter correspond to a sensing area of 4 cm^2^ and were recorded with a sampling period of one second. Figure 3 also displays the thermostat temperature *T*_2_, the ambient temperature *T*_0_, and the calculated temperatures *T*_01_ and *T*_02_. These curves show consistency between the model and the experimental data. As expected, the temperature around the calorimeter, *T*_01_, lies between the thermostat temperature *T*_2_ and the ambient temperature *T*_0_. The low cold-focus temperature *T*_02_ observed in this case corresponds to a Peltier current of *I_pel_* = 0.09 A.

### 2.2. Participants and Anthropometric Measurements

Five young, healthy, male participants, aged between 20 and 23 years, participated in this study. Table 3 shows the anthropometric characteristics of each participant (M1–M5): age, height, body mass, thigh length, maximum thigh circumference, and skinfold thickness. Skinfold thickness was measured using a CX50 ultrasound system (Philips Ultrasound, Inc., Bothell, WA, USA) equipped with an L12-3 linear-array transducer.

### 2.3. Experimental Protocol

All measurements were performed under monitored laboratory conditions during daytime hours. Ambient temperature was 22–24 °C, and relative humidity was 50–60%. The measurement procedures and exercise protocol are described below.

#### 2.3.1. Calorimetric Measurements

**Figure 4:**
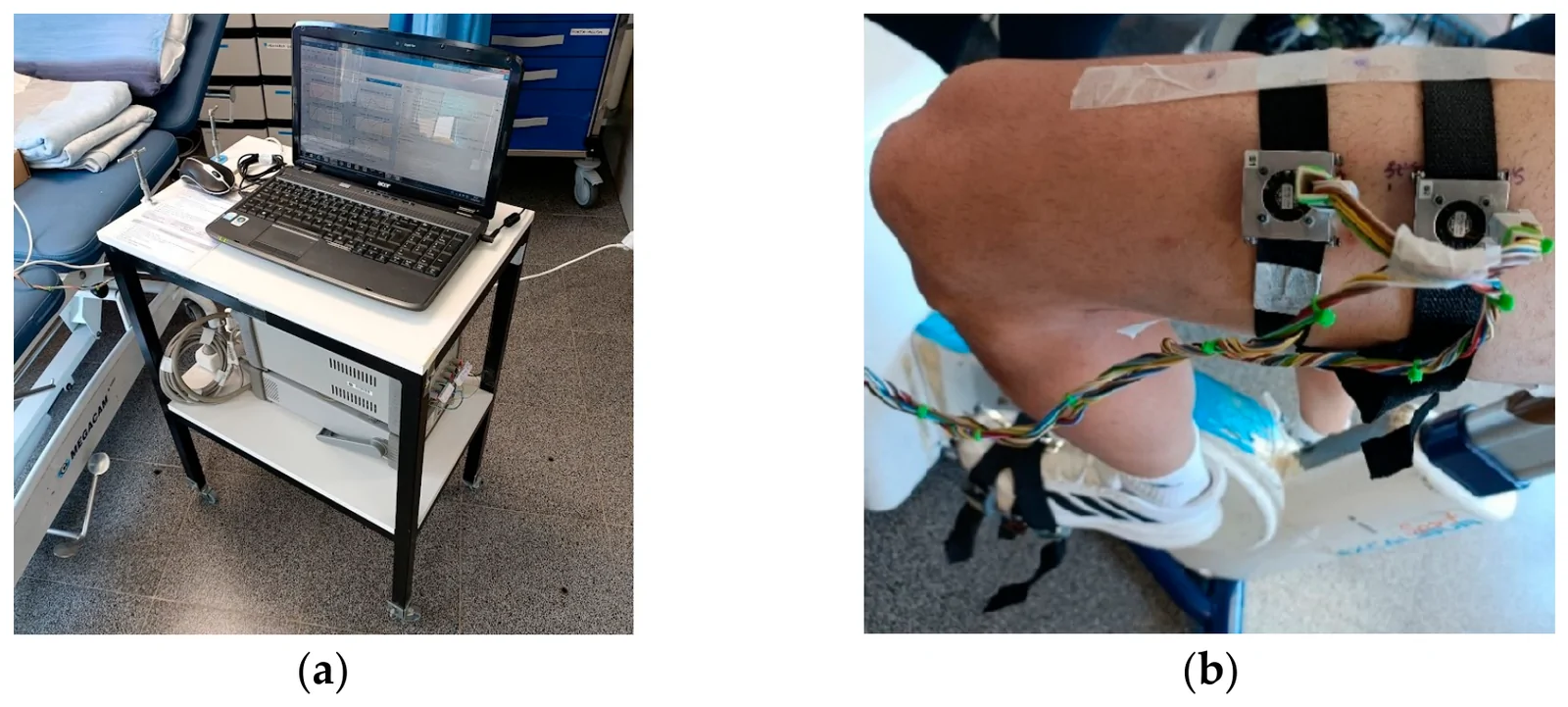
(**a**) Instrumentation used for skin calorimeter operation, including data acquisition, control, and power supply. (**b**) Placement of devices on a participant’s thigh.

Two skin calorimeters were placed over the vastus lateralis muscle to record heat flow before, during, and after the exercise protocol. Figure 4 shows the instrumentation used for skin calorimeter operation, including data acquisition, control, and power supply systems, as well as the placement of the devices on a participant’s thigh. For both skin calorimeters, the thermostat was set at *T*_2_ = 30 °C and the Peltier current to *I_pel_* = 0.1 A throughout all phases of the protocol. At rest, local heat flow decreases as thermostat temperature increases. A thermostat temperature of 30 °C provided a measurement uncertainty of approximately 4% and sufficient resolution for comparisons among participants. Previous exercise measurements performed between 28 and 38 °C showed the highest signal-to-noise ratio at 30 °C [21].

At rest, local heat flow was approximately 150 mW, with a standard deviation of up to 9 mW. Similar fluctuations persisted during exercise, whereas the exercise-related increase in heat flow was approximately 20–80 mW above baseline. Consequently, the signal-to-noise ratio was lower during exercise, making the fluctuations appear larger.

#### 2.3.2. Skin Hydration Measurements

Skin hydration and oil content were measured over the vastus lateralis muscle before, during, and after the exercise protocol, approximately every 2 min. Measurements were performed using a portable skin moisture test analyzer (B077X3WLX9, Brino, China). The instrument is a bipolar bioelectrical impedance skin analyzer with two electrodes separated by 7.1 mm. Its operating principle is based on applying a 3.8 kHz square-wave signal with an amplitude of 0.56 Vpp. The resulting impedance is processed by the device to estimate superficial skin hydration and oil content. The analyzer was used only to qualitatively corroborate the timing of increased skin moisture [35].

#### 2.3.3. Exercise Protocol

**Figure 5:**
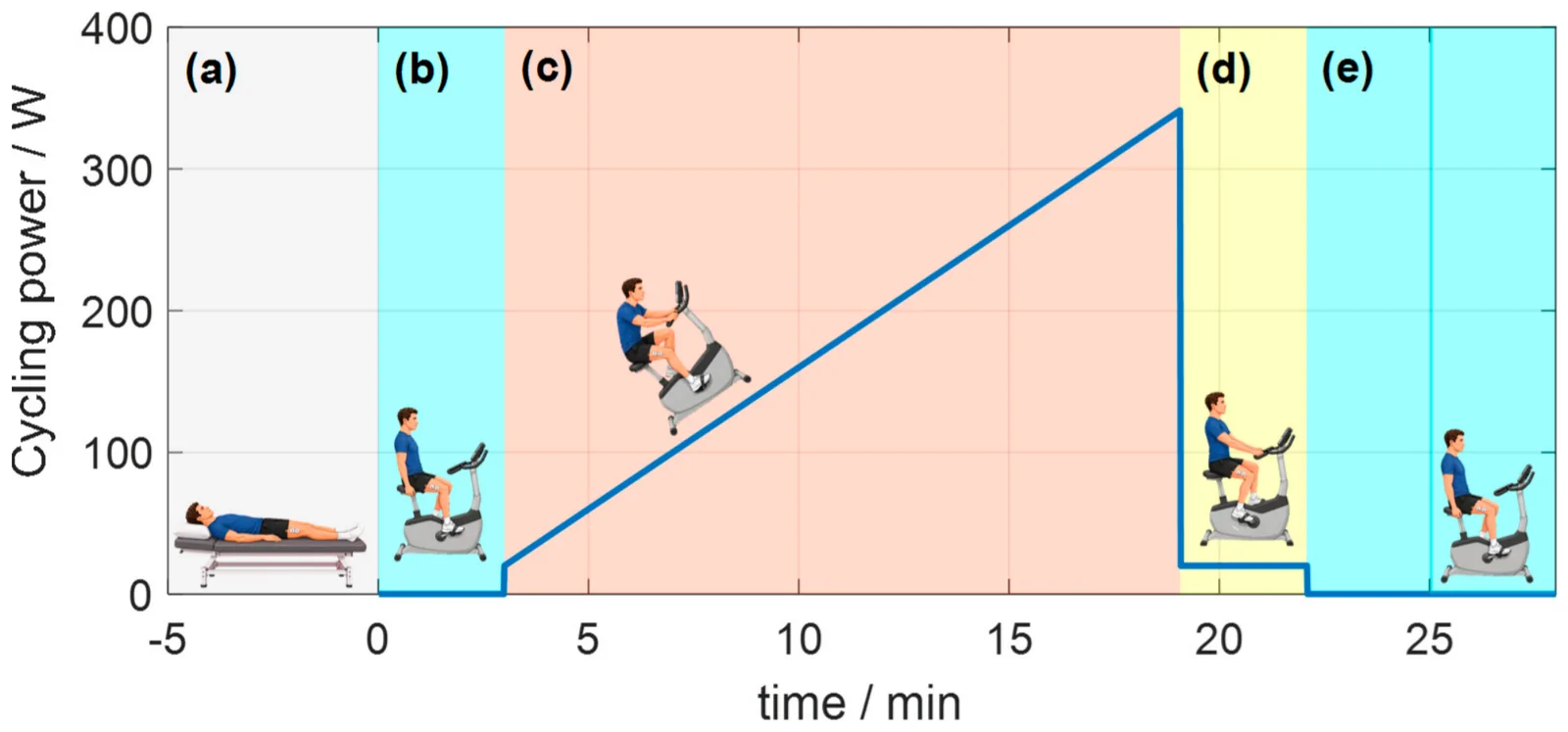
Graphical representation of the exercise protocol: (**a**) participant preparation, (**b**) initial static rest, (**c**) incremental exercise until exhaustion, (**d**) active recovery, (**e**) final static recovery.

Each participant completed two exercise tests on separate days. In each test, calorimetric measurements were obtained at two sites, yielding a total of four calorimetric recordings per participant. Exercise was performed on an Excalibur Sport 925900 cycle ergometer (Lode B.V., Groningen, The Netherlands). The measurement sequence was organized around the exercise protocol, which consisted of the following phases, as shown in Figure 5:

(a) Participant preparation. The participant lay on an examination table while the skin calorimeters were attached to the thigh. Participants wore similar sports clothing, consisting of shorts and a sports shirt.
(b) Initial static rest. The participant was seated on the cycle ergometer and remained without pedaling for 3 min. This period was used to determine the basal heat flow.
(c) Incremental exercise. After resting, the incremental exercise started with a ramp protocol from 20 W, increasing by 20 W min^−1^. Participants were instructed to maintain a pedaling cadence of 80 rpm. Exhaustion was defined as the inability to maintain a cadence above 60 rpm, at which point the exercise ended.
(d) Active recovery. After exhaustion, the workload was reduced to 20 W, and the participant continued pedaling for 3 min.
(e) Final static recovery. The participant remained seated without pedaling for at least 3 min.

**Figure 6:**
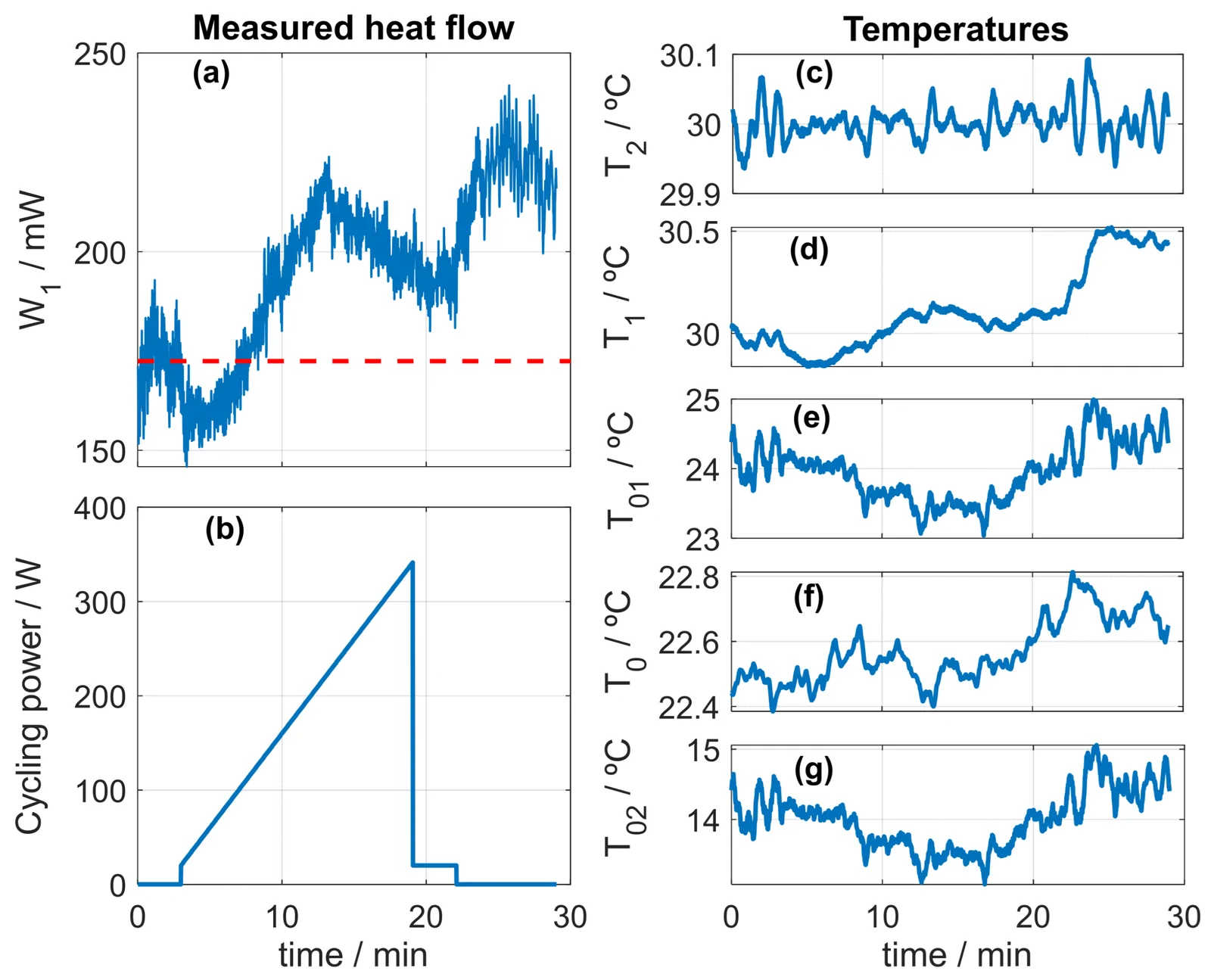
(**a**) Skin heat flow *W*_1_ measured in participant M1 during incremental exercise on a cycle ergometer. The red line represents the resting heat loss, (**b**) mechanical power developed by the participant, calorimeter temperatures *T*_2_ (**c**) and *T*_1_ (**d**), external calorimeter temperatures *T*_01_ (**e**) and *T*_02_ (**g**), and ambient temperature *T*_0_ (**f**).

Figure 6 shows an experiment from participant M1, including the heat flow *W*_1_ and the mechanical power output on the cycle ergometer. In this case, the incremental exercise lasted 16 min. Figure 6 also shows the thermostat temperature *T*_2_ = 30 ± 0.02 °C, the ambient temperature *T*_0_ = 22.6 ± 0.2 °C, and the external calorimeter temperatures *T*_01_ and *T*_02_. *T*_01_ was higher than *T*_0_, consistent with heat released by the participant into the region surrounding the calorimeter. In contrast, *T*_02_ was lower due to the Peltier effect produced at *I_pel_* = 0.1 A. Both *T*_01_ and *T*_02_ decreased slightly during pedaling, an effect attributed to the motion of the calorimeters and the thigh. Finally, the measurement plate temperature *T*_1_ was obtained from the calorimetric signal as *T*_1_ = *T*_2_ + *y*/*k*. *T*_2_ was constant, so *T*_1_ followed the calorimetric signal and showed a pattern similar to the heat flow, but not identical.

### 2.4. Ethical Committee Approval

The study was approved by the Research Ethics Committee of the University of Las Palmas de Gran Canaria (CEIH-2024-02). All participants received detailed information about the experimental procedure and provided written informed consent before participation. The experimental protocol complied with the principles of the Declaration of Helsinki for research involving human participants.

## 3. Results and Discussion

### 3.1. Modeling of Muscle Heat Flow

This section presents the main finding of this work: an analytical thermal model of heat flow in the vastus lateralis during incremental exercise that accurately captures all cases examined in this study. The model includes four main effects:

1. Heating response due to the exercise.
2. Cooling response due to the exercise.
3. Sweating evaporation effect.
4. Recovery overheating effect.

#### 3.1.1. Modeling of Exercise Heating and Cooling Responses

In a previous study [21], the thermal response measured over the rectus femoris was analyzed during a constant-power exercise protocol at 80 W. In that case, the response could be decomposed into two clearly identifiable terms: a rising exponential component and a negative exponential transient that started and ended at zero. For a constant-power exercise protocol, a similar behavior was reported by González-Alonso et al. [36].

In the present study, the exercise protocol is incremental. The local heat flow measured over the vastus lateralis with the skin calorimeter showed a similar pattern in all participants, indicating a consistent thermal response. This finding suggests a functional relationship between the mechanical power produced during exercise and the measured heat flow. An initial decrease in heat flow was also observed at the onset of exercise, followed by a progressive increase. Therefore, the superposition of two terms is considered suitable for describing the measured heat flow during incremental exercise.

For a step input corresponding to a constant mechanical power *W*_0_, the two exponential terms take the form given in Equations (6) and (7), assuming the measurement starts from a steady state corrected to zero.

For a step protocol, with input *W*_0_, in W [21], the response is *y* = *y*_1_ + *y*_2_:

(6) $$y_{1}(t)=A_{1}\left(1-e^{-t/\tau }\right)W_{0}$$

(7) $$y_{2}(t)=\frac{A_{2}}{\tau^{2}}\left(te^{-t/\tau }\right)W_{0}$$

In this study, we assume that the relationship between the mechanical power produced by the participant on the cycle ergometer, considered as the input, and the dissipated heat flow, considered as the output, can be described by a linear differential equation [37]. Under this assumption, the thermal response to an incremental protocol can be derived from the corresponding step response. Because a ramp input, corresponding to incremental exercise, is the time integral of a step input, the response of a linear system to a ramp input can be obtained by integrating the step responses given in Equations (6) and (7). Therefore, for an incremental exercise protocol with slope *W* in W min^−1^, the measured heat flow is described as the superposition of the resulting functions. Equations (8) and (9) give these functions for the case of a zero steady-state initial condition.

For a ramp protocol, input: slope *W*, in W min^−1^ (this work), the response is *y* = *y*_1_ + *y*_2_:

(8) $$y_{1}(t)=\int_{0}^{t}A_{1}\left(1-e^{-t/\tau }\right)Wdt=A_{1}\left(t-\tau +\tau e^{-t/\tau }\right)W$$

(9) $$y_{2}(t)=\int_{0}^{t}\frac{A_{2}}{\tau^{2}}t\left(e^{-t/\tau }\right)Wdt=A_{2}\left(1-e^{-t/\tau }-\frac{t}{\tau }e^{-t/\tau }\right)W$$

**Figure 7:**
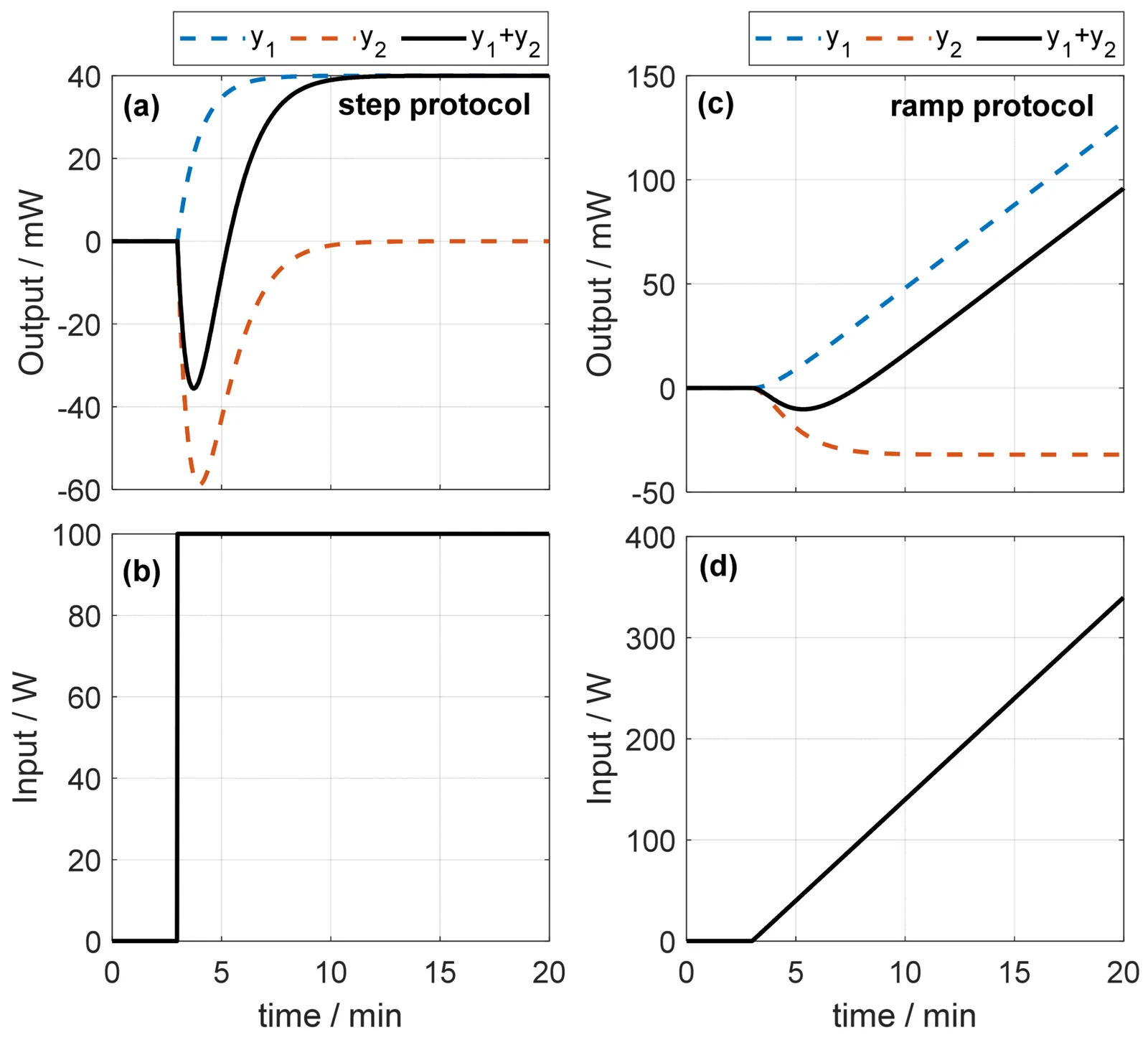
Simulation of the thigh thermal responses for a step exercise protocol and a ramp exercise protocol: (**a**) response to the step protocol, (**b**) step input, (**c**) response to a ramp protocol, (**d**) ramp input. The simulation assumes a zero steady-state initial condition, as described by Equations (6)–(9). The parameters used were *A*_1_ = 0.4 mW·W^−1^, *A*_2_ = −1.6 mW·min·W^−1^, τ = 1 min.

Figure 7 shows these functions for zero initial conditions. The thermal response of the thigh is shown for a constant-power exercise protocol (step input) and an incremental exercise protocol (ramp input). In each case, the response is decomposed into the contributions *y*_1_ and *y*_2_, where *y*_1_ represents muscle heating and *y*_2_ represents a reduction in heat flow, consistent with the initial cooling associated with exercise-induced changes in cutaneous blood flow [38,39,40].

#### 3.1.2. Modeling of Sweating Evaporation

During the ramp protocol, exercise intensity increased progressively until exhaustion, inducing sweating. This was confirmed visually and by an increase in skin moisture measured with the skin moisture test analyzer. Under favorable ambient conditions, evaporation produced local skin cooling [41,42], which was reflected in a decrease in the measured heat flow (see Figure 6a). Whenever evaporation occurred (at the time *t_eva_*), this decrease in heat flow was well described by Equation (10), a linear fit with a negative slope:

(10) $$y_{3}(t)=A_{3}(t-t_{eva})$$

Over the short interval during which evaporation occurred, the model adequately reproduced the experimental response. This simplified approach may serve as a starting point for more detailed future studies. The onset of sweat evaporation, *t_eva_*, was determined by visual inspection of the heat flow curve as the point at which a clear change in slope appeared, consistent with the onset of evaporative heat loss. The same criterion was applied to all records. Future versions of the skin calorimeter could incorporate electrical bioimpedance to simultaneously measure skin moisture, in line with current trends toward multimodal devices [43].

#### 3.1.3. Modeling of Recovery Overheating

After exercise ends, the participant remains on the cycle ergometer and continues pedaling at a load of 20 W for 3 min. When pedaling stops, the heat flow increases abruptly (see Figure 6a). This response is modeled with a function of the same type as *y*_2_ (Equation (7)), which represents the response to an inverted step input. This effect has been observed in our previous studies [21] and is thought to be primarily attributable to changes in blood flow. This interpretation is consistent with previous studies on human thermoregulation, limb perfusion, and muscle temperature during and after exercise [6,8,9,36], as well as with specific studies on post-exercise heat balance and hyperemia [44,45].

### 3.2. Experimental Results

Now, we describe in detail the fitting of a complete measurement obtained from participant M1. The parameters *A*_1_, *A*_2_, and τ of these functions were estimated by minimizing the RMSE between the heat flow curve and the modeled curve described by Equations (8) and (9), using the same Nelder–Mead-based algorithm described above [28,29,30].

**Figure 8:**
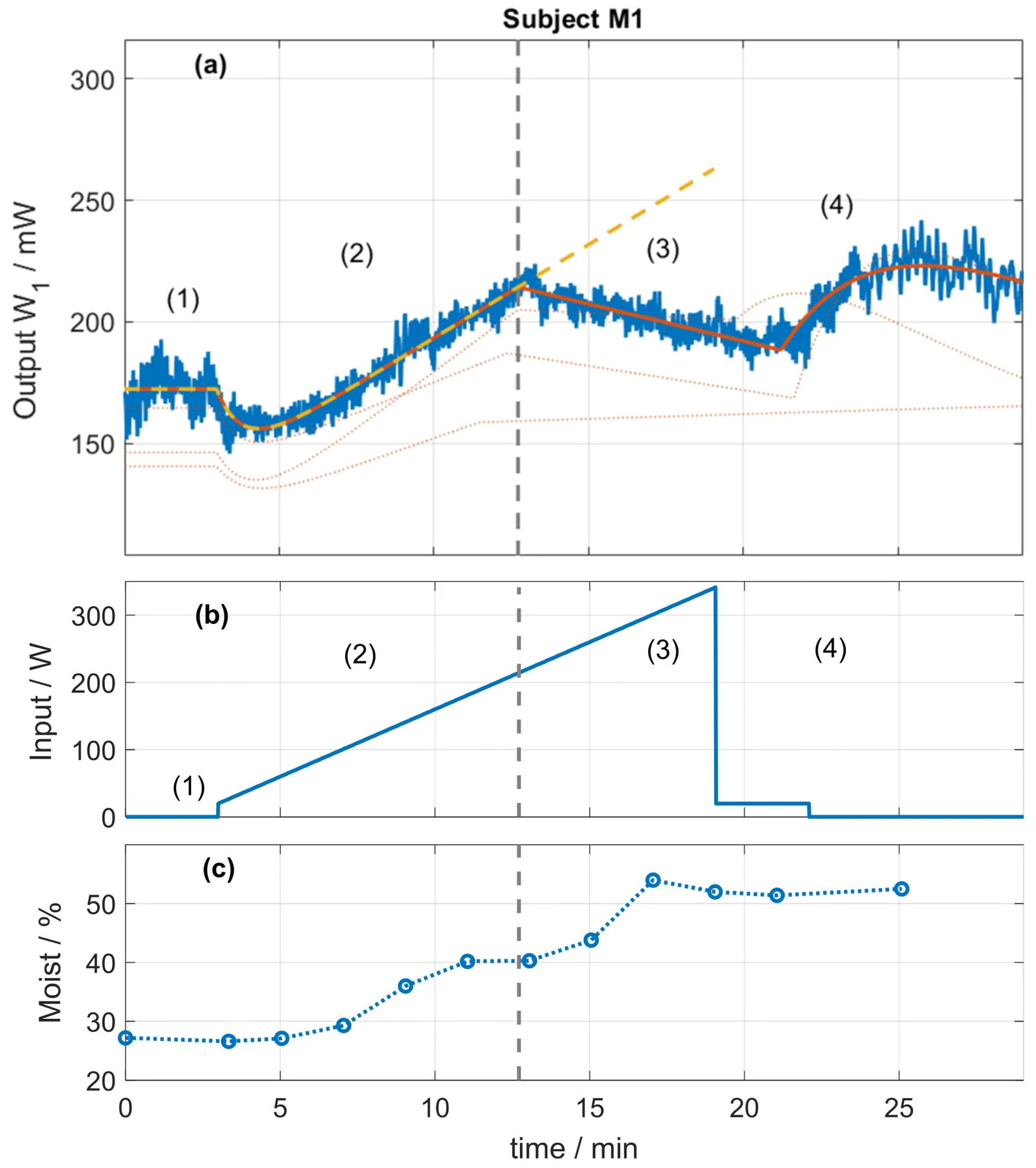
Participant M1 measurement. (**a**) Fitting of the heat flow curve. The fitted curve is shown as a continuous red line, and the measured heat flow is shown in blue. The parameters obtained were *A*_0_ = 172.5 mW, *A*_1_ = 0.38 mW·W^−1^, *A*_2_ = –1.68 mW·min·W^−1^, t = 0.90 min, and *A*_3_ = –10.72 mW·min^−1^. For the recovery segment, *A*_4_ = –1.85 mW·min·W^−1^ and τ*_rec_* = 5.21 min. RMSE = 6 mW. (**b**) Mechanical power produced by the participant on the cycle ergometer is labeled as input. (**c**) Local skin moisture (%).

The measurement, displayed in Figure 8, was decomposed into four segments, as explained at the beginning of the Section 3: (1) heating response induced by exercise, (2) cooling response induced by exercise, (3) cooling effect due to sweat evaporation, and (4) overheating during recovery.

1. In the first segment, the participant is seated on the cycle ergometer at rest. This segment lasts 3 min and represents the initial steady state, with a heat flow *A*_0_. For the case shown in Figure 8, *A*_0_ = 172.5 mW.

(11) $$y(t)=A_{0}$$

2. In the second segment, the participant performs incremental exercise. The heat flow curve was fitted using the functions *y*_1_(t) and *y*_2_(t) given by Equations (8) and (9);. The time function for this curve segment is:
  (12) $$y(t)=A_{0}+A_{1}(t-\tau +\tau e^{-t/\tau })W+A_{2}\left(1-e^{-t/\tau }-\frac{t}{\tau }e^{-t/\tau }\right)W$$
  where the fitted parameters are *A*_1_ = 0.38 mW·W^−1^, *A*_2_ = −1.68 mW·min·W^−1^, τ = 0.9 min and *W* = 20 W min^−1^.

3. In the third segment, the participant is still exercising, but evaporation begins to affect the measured heat flow. If evaporation starts at time *t_eva_* (see moist curve shown in Figure 8c), the time function for this curve segment is, for *t* > *t_eva_*:
  (13) $$y(t)=A_{0}+A_{1}(t-\tau +\tau e^{-t/\tau })W+A_{2}\left(1-e^{-t/\tau }-\frac{t}{\tau }e^{-t/\tau }\right)W+A_{3}(t-t_{eva})$$
  the parameter *A*_3_ is introduced to represent the slope associated with the cooling produced by evaporation. In this case, *A*_3_ = −10.72 mW min^−1^.

4. After exercise, the participant continues pedaling at 20 W for 3 min. Once pedaling stops, an additional recovery term, similar to *y*_2_ in Equation (7), is included in the heat flow function. The resulting time function for this segment is:
  (14) $$y(t)=A_{0}+\left[y_{0}-A_{0}+\left({\left(\frac{dy}{dt}\right)}_{0}-u_{0}\frac{A_{4}}{\tau_{rec}^{2}}+\frac{y_{0}-A_{0}}{\tau_{rec}}\right)t\right]e^{-t/\tau_{rec}}$$
  where *u*_0_ is the final mechanical power, *y*_0_ is the heat flow at the end of exercise, and (*dy/dt)*_0_ is its time derivative. For the final recovery segment shown in Figure 8, the fitted parameters are *A*_4_ = −1.85 mW·min·W^−1^ and τ*_rec_* = 5.25 min. Note that this segment has a different time constant τ*_rec_*.

**Figure 9:**
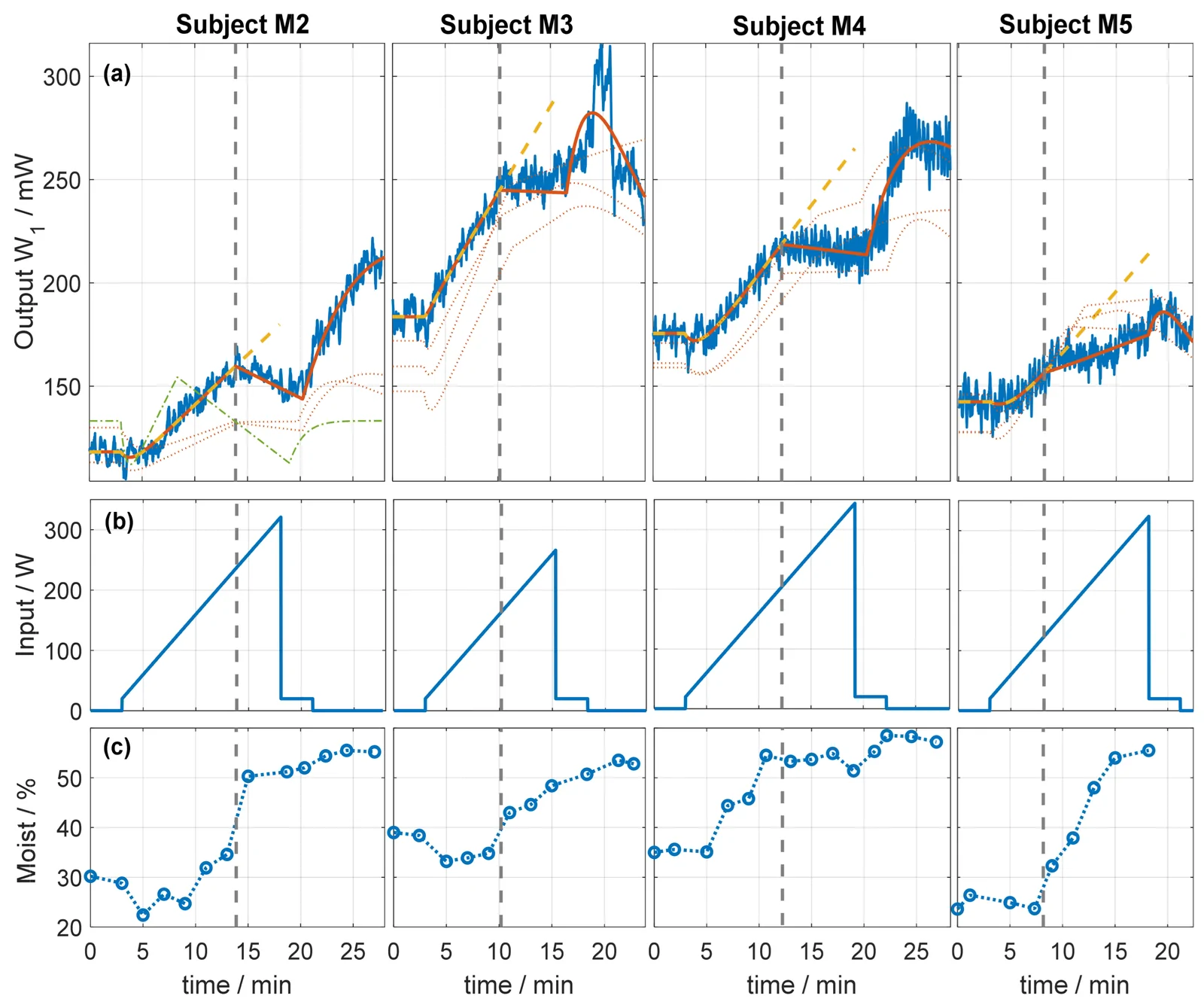
Thermal response and model fitting for participants M2, M3, M4, and M5. (**a**) Heat flow measurements and corresponding fitted curves for each participant. The measured heat flow *W*_1_ is expressed in mW. (**b**) Mechanical power developed by the participant on the cycle ergometer, used as the input signal for the heat flow model and expressed in W. (**c**) Skin moisture measurement, expressed as moisture percentage (%).

With the model parameters defined, we now present results for the other four participants. Figure 9 shows the thermal behavior of participants M2 to M5, whereas Figure 8 shows that of participant M1. Both figures show agreement between the heat flow measured with the skin calorimeter and the thermal model’s functions throughout the complete protocol. In both Figure 8 and Figure 9, the response is decomposed into the four contributions described previously, physiologically interpreted as heating associated with muscle metabolic activity, cooling associated with changes in blood perfusion, cooling due to sweat evaporation, and final overheating during recovery. The onset of evaporation is indicated by a vertical dashed line and coincides with an increase in local skin moisture, marking the point at which the evaporation term is included in the model. Although additional measurements are included in Figure 8 and Figure 9, shown as small red dotted lines, only the final fitted function is displayed. The comparison between the experimental measurement and the model fit is presented for one representative measurement from each participant.

The numerical values of the fitted parameters are summarized in Table 4, which reports the mean values and standard deviations for each participant (M1–M5) and for the different time segments analyzed: rest, incremental exercise, evaporation, and post-exercise. The table includes the characteristic model parameters *A*_0_, *A*_1_, *A*_2_, *A*_3_, *A*_4_*,* the time constants for the exercise (τ) and post-exercise segments (τ*_rec_*), as well as the fitting error RMSE. Together, these parameters provide a compact description of the individual thermal responses and allow comparison of the main model contributions across participants.

### 3.3. Discussion

Although the thermal response varied among participants, the fitted parameter remained within relatively limited ranges, suggesting that the observed differences mainly reflect intersubject variability. The following discussion analyzes the meaning of each model parameter. Table 5 summarizes the main correlations found for these parameters. Given the limited number of participants, these correlations should be considered exploratory.

#### 3.3.1. Parameters A_0_, A_1_ and A_2_

The parameter *A*_0_ represents the resting heat flow. It is relatively homogeneous across participants. The overall mean value was 150 ± 20 mW. Consistent with the Pennes equation framework [16,17,18], resting heat flow is expected to depend on local tissue properties and geometry of the thigh, which was approximated as a cylinder with volume *V* and surface area *S*. *A*_0_ was found to be inversely associated with skinfold thickness *e*, and positively associated with the surface to thickness ratio (*S/e*). These results are consistent with the expected effect of superficial tissue thickness on conductive heat transfer, and with previous studies developed by other authors [46,47,48,49].

**Table 5:** Main correlations found for parameters *A*_0_ (basal heat flux), *A*_1_ (positive muscle heating term), *A*_2_ (negative muscle heating term) and Δ = τ − *A*_2_/*A*_1_ (effective delay) with skinfold thickness (*e*), surface to thick ratio (*S/e*), height (*H*), mass (*M*), volume (*V*) and volume to surface ratio (*V/S*). All correlations are exploratory and correspond to the mean values for each participant (n = 5).

Correlation	*r*	*p*-Value	Meaning	Ref.
*A*_0_ vs. *e*	−0.742	0.1510	More *e*, less basal dissipation	[46,48,49]
*A*_0_ vs. *S/e*	0.757	0.1390	More *S/e*, more basal dissipation	[16,46,48]
$\bar{A}$_1_ vs. *V/S*	−0.806	0.0996	More *V/S*, less dissipation rate	[50]
*σA*_1_ vs. *e*	0.909	0.0324	More *e*, more dissipation rate variability	[46,48,49]
$\left|{\bar{A}}_{2}\right|$ vs. *H*	0.895	0.0400	More *H*, more exercise induced cooling	[8,38,39,50]
$\left|{\bar{A}}_{2}\right|$ vs. *M*	0.890	0.0431	More *M*, more exercise induced cooling	[8,38,39,50]
Δ vs. *V/S*	0.926	0.0240	More *V/S*, more thermal inertia	[50]
Δ vs. *V*	0.895	0.0401	More *V*, more thermal inertia	[50]

The parameter *A*_1_ reflects the sensitivity of heat flow to the increase in mechanical power during exercise. This parameter showed some intersubject variability, which may be related to differences in metabolic efficiency, local heat-transfer geometry, or heat-dissipation capacity. In this sense, *A*_1_ showed a negative association with the thigh volume-to-surface ratio (*V*/*S*), suggesting lower sensitivity in participants with larger thigh size [50]. As shown in Figure 8 and Figure 9, the measured signals followed similar overall trends across participants, although some differences in curve shape were observed. The main exception was one measurement from participant M2, shown as the green curve in Figure 9a. In this case, evaporation began earlier than in the other measurements, thereby modifying the heat-flow response during the incremental exercise segment. This atypical behavior explains the larger standard deviation of *A*_1_ for participant M2 in Table 4. In the remaining participants, the dispersion of *A*_1_ was lower. Overall, the standard deviation of *A*_1_ was positively associated with skinfold thickness *e*, suggesting that a thicker superficial tissue layer may reduce the reproducibility of the exercise-heating coefficient [46,48,49].

The negative parameter *A*_2_ was interpreted as the cooling component observed at the onset of exercise, which is consistent with exercise-induced changes in skin and muscle perfusion [8,14,15,38,39]. This phenomenon contributes to the delay between mechanical input and the thermal heat flow response. Larger participants tended to show higher *A*_2_ values, suggesting that the blood-flow-related cooling term may scale with participant size, consistent with previous work on the influence of body morphology on heat exchange [50].

In the phase where evaporation appeared, the parameter *A*_3_ introduced an additional cooling contribution, consistent with heat loss by sweating [41,42]. The onset of evaporation, indicated in Figure 8 and Figure 9 by a vertical dashed line, coincided with an increase in skin moisture, supporting the inclusion of this term in the model.

On the other hand, after exercise cessation, the measured heat flow showed an abrupt recovery overshoot, with variable shapes across participants and measurements. The parameter *A*_4_ accounts for this post-exercise increase, which is consistent with altered heat balance and changes in local blood supply during recovery [44,45].

#### 3.3.2. Time Constants, τ and τ_Rec_

Finally, the time constants reflected the dynamics of the thermal system, showing a faster response during exercise (τ ≈ 1 min) than during recovery (τ ≈ 6 min). In the incremental exercise phase, the response to a ramp input tends to a ramp with a temporal delay, which can be expressed as Δ = τ − *A*_2_/*A*_1_, with a mean value of 4 ± 1 min. Δ combines the intrinsic time constant, the exercise-heating coefficient, and the blood-flow-related cooling term, thereby providing a more complete descriptor of the participant’s thermal inertia. Δ was found to increase with thigh volume (*V*) and with the volume-to-surface-area ratio (*V/S*), further suggesting that thermal inertia is related to thigh size [50].

#### 3.3.3. Limitations

Measurement duration is an important factor in reliable parameter estimation. During the incremental exercise phase, approximately 15 min of recording captured the main components of the thermal response. However, the recovery phase was shorter and had to be described with a single simplified function. Future protocols with longer post-exercise recordings may allow more detailed characterization of recovery dynamics.

Another limitation is the sample size. In our previous work [21], only one participant was analyzed. In the present study, the sample was increased to five participants. This design allows some trends to be explored, but the statistical significance remains limited. Even so, the consistency of the observations reported here with previous studies [46,47,48,49,50], together with the analytical relationship with the previous model [21], supports the value of these measurements and the robustness of the thermal model presented in this work. Regarding sweat evaporation, this phenomenon was incorporated into the model as an additional cooling term. However, if evaporation occurs too early, it may affect the measured heat-flow signal and modify the fitted response. Finally, although the calorimeters were placed over the vastus lateralis at positions selected to target active muscle regions, small differences in local tissue composition may have influenced the measured heat flow.

## 4. Conclusions

This study demonstrates that skin calorimetry is a feasible non-invasive approach to characterizing local thermal dynamics in the vastus lateralis muscle during incremental exercise. The measured heat flow curves were accurately reproduced using the thermal model proposed, with an RMSE of 6.7 ± 0.8 mW for a 2 × 2 cm^2^ sensing area.

- The main result of this work is the analytical continuity between the present incremental-exercise thermal model and the previous constant-power formulation. Since a ramp increase in mechanical power is the time integral of a power step, the corresponding heat-flow response can be obtained by integrating the step-response expressions.
- The model separated the measured heat flow into five contributions: resting heat dissipation *A*_0_, and four contributions physiologically interpreted as exercise-induced heating *A*_1_, blood-flow-related cooling *A*_2_, evaporative cooling *A*_3_, and post-exercise overheating during recovery *A*_4_.
- At rest, *A*_0_ was 150 ± 19 mW. During exercise, *A*_1_ was 0.36 ± 0.14 mW·W^−1^, with a time constant of 1.00 ± 0.32 min. The cooling effect *A*_2_ produced a drop of −20 mW for the 20 W·min^−1^ ramp protocol. In the later stages of exercise, the evaporative cooling term was *A*_3_ = −7.06 ± 2.98 mW min^−1^. Finally, after exercise, recovery shows slower dynamics, with τ_rec_ = 6.47 ± 2.63 min. These parameters showed several correlations consistent with the literature, related to thigh geometry and skinfold thickness.
- In addition, the effective delay parameter Δ = τ − *A*_2_/*A*_1_ served as a compact descriptor of thermal inertia and was also associated with thigh geometry.

Overall, these results indicate that local skin calorimetry can decompose and quantify distinct thermal contributions during exercise and recovery. However, this study is limited by the small sample size, the short duration of the recovery recordings, and the lack of complementary physiological measurements.

## Figures and Tables

**Table 1:** Parameters of the calorimetric model (Equations (2) and (3)), for the two skin calorimeters, denoted as S1 and S2. Mean values and standard deviations (std) are shown.

Parameter	Units	S1 (mean)	S1 (std)	S2 (mean)	S2 (std)
*C*_1_	J·K^−1^	2.5680	0.0630	2.3110	0.0460
*C*_2_	J·K^−1^	3.6280	0.0470	3.5630	0.0890
*P*_1_	W·K^−1^	0.0300	0.0007	0.0285	0.0008
*P*_12_	W·K^−1^	0.0907	0.0018	0.0918	0.0017
*k*	V·K^−1^	0.0234	0.0004	0.0241	0.0003
*P*_2_ *= a + b·I_pel_* ^1^	W·K^−1^	**S1**	**RMSE**	**S2**	**RMSE**
*a*	W·K^−1^	0.0533	0.0008	0.0521	0.0012
*b*	W·K^−1^·A^−1^	0.0295	0.0008	0.0225	0.0012

^1^ *P*_2_ value depends on the Peltier current *I_pel_*.

**Table 2:** Parameters of the thermal model describing the external temperature difference (Equations (4) and (5)). Expanding the relation in Equation (5) gives: Δ*T* = *T*_01_ − *T*_02_ = α_0_ + α_1_*·I_pel_* + α_2_*·I_pel_*^2^.

Parameter	Units	S1	S2
*α*_0_	°C	−0.0427	−0.5230
*α*_1_	°C·A^−1^	108.20	111.37
*α*_2_	°C·A^−2^	−81.390	−82.550
RMSE (Δ*T*)	°C	0.0627	0.0563

**Table 3:** Anthropometric characteristics and thigh measurements of the participants.

Participant	Unit	M1	M2	M3	M4	M5
Age	years	20	23	22	21	23
Height ***H***	m	1.90	1.77	1.73	1.72	1.73
Mass ***M***	kg	90	79	64	75	56
Thigh length ^1^ ***L***	cm	50.7	47.5	48.0	47.0	48.0
Thigh circumference ***C***	cm	62.8	64.3	52.1	55.0	54.3
Skinfold thickness ***e***	mm	6.0	7.5	3.6	2.7	4.2

^1^ Distance between the iliac crest and the lateral femoral condyle.

**Table 4:** Mean values ± standard deviations of the fitted parameters obtained from the heat flow measurements for each participant (M1–M5). The parameters are grouped according to the time segments used in the model: (1) initial rest, corresponding to the initial steady state; (2) incremental exercise at 20 Wmin^−1^, described by Equation (12); (3) continued incremental exercise with sweat evaporation, described by Equation (13); and (4) post-exercise recovery, described by Equation (14). The table also reports the fitting error RMSE for each participant. (n = 20).

	M1	M2	M3	M4	M5	Mean
Parameter		(1) Initial rest				
*A*_0_ ^1^	156 ± 15	124 ± 9	166 ± 16	167 ± 8	136 ± 10	150 ± 20
Parameter		(2) Incremental exercise				
*A*_1_	0.33 ± 0.12	0.24 ± 0.19	0.52 ± 0.10	0.34 ± 0.05	0.37 ± 0.06	0.36 ± 0.14
*A*_2_	−1.40 ± 0.32	−0.91 ± 0.62	−0.73 ± 0.44	−1.00 ± 0.45	−0.77± 0.29	−0.96 ± 0.46
τ	0.94 ± 0.07	0.89 ± 0.17	0.62 ± 0.14	1.38 ± 0.27	1.15 ± 0.29	1.00 ± 0.32
Parameter		(3) Sweat evaporation during exercise				
*A*_3_	−7.94 ± 3.03	−6.55 ± 5.52	−8.39 ± 2.05	−5.74 ± 2.28	−6.67 ± 1.04	−7.06 ± 2.98
Parameter		(4) Post-exercise recovery				
*A*_4_	−1.99 ± 0.07	−5.49 ± 4.78	−0.48 ± 0.63	−2.40 ± 1.31	−0.55 ± 0.66	−2.18 ± 2.03
τ*_rec_*	4.76 ± 1.10	9.70 ± 2.99	5.95 ± 2.99	6.69 ± 1.75	5.26 ± 1.87	6.47 ± 2.63
*RMSE*	6.7 ± 0.7	5.6 ± 0.8	7.6 ± 0.8	7.0 ± 1.3	6.3 ± 0.7	6.7 ± 0.8

^1^ *A*_0_ in mW, *A*_1_ in mW·W^−1^, *A*_2_ and *A*_4_ in mW·min·W^−1^, *A*_3_ in mW·min^−1^ and RMSE in mW.

## Data Availability

All data underlying the results are available as part of the article, and no additional source data are required.

## Abbreviations

The following abbreviations are used in this manuscript:

DHF	Dual Heat Flux
PID control	Proportional–Integral–Derivative Controller
RMSE	Root Mean Square Error
EPS	Expanded Polystyrene
Rpm	Revolutions per minute
Std	Standard deviation
Vpp	Peak-to-peak voltage
